# Prognostic Factors for Mortality in Adults Hospitalized With COVID-19 Infection in the Americas

**DOI:** 10.7759/cureus.55044

**Published:** 2024-02-27

**Authors:** Poonam Tawde, Loveth Igburuke, David Olanipekun, Vansh Marwaha, Jonathan Lambo

**Affiliations:** 1 Internal Medicine, Avalon University School of Medicine, Willemstad, CUW; 2 Internal Medicine, Mountain Vista Medical Center, Mesa, USA; 3 Epidemiology and Public Health, Avalon University School of Medicine, Willemstad, CUW

**Keywords:** sars-cov-2, patient characteristics, disease severity, mortality, cohort study, hospitalized patients, demographics, age, covid 19

## Abstract

Many reports on COVID-19 have highlighted an association between patient characteristics upon admission for COVID-19 and how they affect variable progressions of the disease severity and, ultimately mortality.

In this cohort, we analyzed data from patients in the Americas who tested positive for severe acute respiratory syndrome coronavirus 2 (SARS-CoV-2), the virus that causes COVID-19, and were hospitalized. We retrieved data from identified studies selected from full-text screening to identify relevant patient demographics: age, gender, race, duration of hospital stay, and comorbidities. Data were assessed for consistency, missing values, presence of outliers, and implausible values were documented. Our analysis was performed by calculating the case fatality rates for data reported by the original authors within the studies. The primary outcome was mortality.

Participants in the review were COVID-19 inpatients diagnosed through reverse-transcriptase polymerase chain reaction tests (RT-PCR) and above the age of 45 years in the North and South American continents. The study included 12,895 patients who were hospitalized with the COVID-19 infection, and an overall 14.4% in-hospital case-fatality rate was demonstrated. The prognostic factor with the most significant association for increased risk of in-hospital mortality in the elderly was having a high viral load of the COVID-19 virus with a case-fatality rate of 41.4%. In addition, being of Caucasian ethnicity and having the comorbidity of chronic obstructive pulmonary disease (COPD) were also associated with higher rates of mortality among elderly hospitalized patients with case-fatality rates of 40.8% and 35.7% respectively.

Prognostic factors for COVID-19 mortality were determined by having a high viral load of the infection, being of Caucasian ethnicity, and having underlying pulmonary comorbidities such as COPD. In the future research should be undertaken to determine the underlying mechanisms behind these effects.

## Introduction and background

The novel beta coronavirus, severe acute respiratory syndrome coronavirus 2 (SARS-CoV-2), the causative agent of COVID-19, first appeared in Wuhan, Hubei Province, China in December 2019 [1]. It has led to a global pandemic with over 254 million people infected worldwide, 95 million of those from the Americas, with an overall mortality rate ranging between 1.6% and 5% [2-3]. While the disease is mild and self-limiting in many people, in a considerable number of patients, the disease is severe and fatal [4]. Symptomatic COVID-19 infection usually presents as a respiratory syndrome, most commonly ranging from a mild common flu-like disease to severe pneumonia [5]. One of the largest cohorts of patients with COVID-19 showed that around 15% typically need hospital care, and another 5% have a critical illness requiring more intensive support [5]. Prior reports identified that patients with certain comorbidities are more likely to present to the hospital. Furthermore, older age, higher Sequential Organ Failure Assessment (SOFA) score, and elevated d-dimers were significantly associated with an increased risk of inpatient mortality [6]. More recent data show that the median age of U.S. COVID-19 patients was 48 years, with the elderly more susceptible to SARS-CoV-2, and 70% of them are more likely to progress to severe or critical illness [7]. Patients aged ≥ 80 years typically present with low oxygen saturation at admission and high c-reactive protein and elevated lactate dehydrogenase while hospitalized, which are associated with rapid progression to death [8]. Although younger adults are less likely than older adults to develop severe infections, 5% of all severe cases aged ≤ 50 years develop serious symptoms and complications, including severe pneumonia and, more rarely, encephalitis and cardiovascular diseases [9]. The objective of the study is to assess the prognostic factors that are associated with a high risk of mortality in hospitalized elderly patients with COVID-19 infection in the Americas. We ask whether prognostic factors such as gender, race, and other co-morbidities play a significant role in affecting in-hospital mortality and support the design of further trials. In this study, we evaluate data on cases of COVID-19 in patients aged ≥ 45 years hospitalized in the Americas and analyze the various characteristics upon admission associated with in-hospital death to provide clinical data to aid in understanding the pathophysiology of this disease.

## Review

Methodology

Search Strategy

The PubMed database was searched exclusively for prospective and retrospective cohort study designs from 2019-2021. We combined the following search terms from themes/concepts: 1. adult(s) 2. COVID-19 3. prognostic factors 4. mortality. Search terms were combined as free words, keywords, or synonyms. The search strategy used within this research was combined in order of the requisite population of adult populations above the age of 45 years old, the exposure to the COVID-19 infection, and the outcome which was prognostic factors for the infection given various demographics and/or comorbidities. The strategy employed for searching the PubMed database involved the use of the following terms (aged/elder*/senior(s)/adult*') and (COVID-19/SARS-CoV 2/SARS-CoV 2 variants) and (mortality/prognostic factor(s)/prognos*/prognosis/prediction/follow-up/predic*). The asterisk (*) was strategically employed as a wildcard character to account for variations in word endings and to broaden the scope of the search. We included the filters 'middle-aged' and 'aged 45+ years' in the study design to create a systematic review of observational studies. The citations retrieved were initially exported and subsequently screened based on their titles and abstracts. Any disagreements were resolved through consensus.

Study Selection and Eligibility Criteria

Full-text articles of the citations of the studies that met the abstract and title screening were screened using the population, intervention, exposure, and outcome (PECO) concept [10]. The inclusion and exclusion criteria for the study are described in (Table 1).

**Table 1:** Inclusion and exclusion criteria.

Criteria	1. Patient Characteristics	2. Patient Outcome	3. Study Design	4. Language
Inclusion	Age > 45, clinically tested and diagnosed with COVID-19, hospitalized for the condition	Mortality, risk factors, incidence rate, person-time at risk, case fatality	Observational study (retrospective or prospective cohort)	English
Exclusion	Hospitalized for conditions other than COVID-19 and/or age < 45	-	Review articles, case studies, qualitative studies, narrative reports, guidelines, reports describing protocols, unpublished articles, grey literature, theses, dissertations	Languages other than English

Measurement and Observation: Outcome, Exposure, Covariates

The primary outcome measure was mortality in the hospital regardless of time. We used case fatality rates (CFRs) based on the following selected prognostic factors: age, gender, race, and socioeconomic status as a measure of mortality (Table 2). The CFR is a clinical measure of the severity of disease and was calculated from the reported number of COVID-19 deaths as a proportion of the total COVID-19 hospitalized cases per study. Case fatality was chosen as a measure of prognosis over the mortality rate, as mortality represents the entire population at risk of dying, while case fatality is limited to those who already have the disease. Therefore, this ensures it is a better measure of disease severity. Additionally, the case-fatality rate is suited to diseases that are short-term and acute conditions such as COVID-19 infection and is less useful in chronic diseases [11].

**Table 2:** Characteristics of studies included in a systematic review of prognostic factors for COVID-19 in hospitalized elderly patients. HTN: hypertension, CT: cycle threshold, COPD: chronic obstructive pulmonary disease, ESRD: end-stage renal disease, NSAIDs: nonsteroidal anti-inflammatory drugs, ACEi/ARB: angiotensin-converting enzyme inhibitors/angiotensin receptor blockers, BMI (iqr): body mass index (interquartile range)

Author's Name	Number of Patients	Male Deaths (%)	Female Deaths (%)	Male Cases (%)	Female Cases (%)	Other Prognostic Variables	Outcomes
Eboni et al. [12]	1,382	NA	NA	48.9	51.1	Race (Black vs Caucasian patients)	Black - 230 deaths (1,063 patients), Caucasian - 96 deaths (319 patients).
Bode et al. [13]	1,122	NA	NA	55.6	44.4	Presence of Diabetes Mellitus/Uncontrolled Hyperglycemia	Presence of Diabetes Mellitus- 130 deaths (451 patients), Absence of Diabetes Mellitus- 41 deaths (671 patients).
Garassino et al. [14]	200	NA	NA	29.5	70.5	Thoracic Malignancies	Presence of Thoracic Malignancy - 66 deaths (200 patients).
Wang et al. [15]	3,273	26.4	23.4	57.3	42.7	Race, Hypertension, Obesity, Diabetes, HIV, Malignancies	HTN - 314 deaths (1,082 patients), Obesity - 56 deaths (261 patients), Diabetes Mellitus - 213 deaths (768 patients), HIV - 10 deaths (52 patients), Malignancy - 62 deaths (233 patients).
Choudhuri et al. [16]	1,044	29.8	20.9	55.6	44.4	COVID-19 Cycle Threshold (Viral Load) - Low CT means higher viral load, Diabetes, Hypertension	Q1 (<22.9) - 109 deaths (263 patients), Q2 (23.0 - 27.3) - 84 deaths (263 patients), Q3(27.4 - 32.8) - 43 deaths (260 patients), Q4(>32.9) - 34 deaths (258 patients), Diabetes - 139 deaths (581 patients), Hypertension - 193 deaths (629 patients).
Berry et al. [17]	3,123	14.44	8.38	76.07	31.21	Comorbidities, Respiratory Rate, Oxygenation less than 94, Asthma, COPD, Diabetes, Insulin Use, Bradycardia on admission, Hypertension, Hypotension on admission, Coronary Disease, Stroke, Heart Failure, Arrhythmia, Cancer, Renal Failure, Advanced Liver Disease, Rheumatologic Disorder, HIV or Hepatitis, Inflammatory Bowel Disease, Current or former Smoking, Non-Smoking	Respiratory Rate -169 deaths (373 patients), Oxygenation less than 94 - 394 deaths (1,270 patients), Asthma 58 deaths (276 patients), COPD 91 deaths (221 patients), Diabetes 286 deaths (943 patients), Insulin Use 186 (581 patients), Bradycardia on admission 42 (62 patients), Hypertension 52 (1649 patients), Hypotension on admission 172 (491 patients), Coronary Disease 177 (448 patients), Stroke 63 (145 patients), Heart failure 107 (227 patients), Arrhythmia 125 (269 patients), Cancer 122 (357 patients), Renal Failure 101 (216 patients), Advanced Liver Disease 12 (26 patients), Rheumatologic Disorder 31 (89 patients), HIV or Hepatitis 38 (151 patients), Inflammatory Bowel Disease 7 (25 patients), Current 19 (113 patients), Former 161 (539 patients), Non-smoking 414 (2068 patients).
Chaudhary et al. [18]	128	N/A	26.56	N/A	21.87	Comorbidities, Race, BMI, kg/m2, Bilateral infiltrates, Respiratory, COPD, Asthma, Obstructive sleep apnea, Cardiovascular, Heart failure, Atrial fibrillation, Coronary Disease, Hypertension, Liver, Cirrhosis, Liver transplant, Renal, Chronic kidney disease, ESRD on dialysis, Kidney transplant, Other, Diabetes, HIV, Medications at admission, Antiplatelets, NSAIDS, ACEi/ARB	Race: African-American 53 deaths (82 patients), Black 53 deaths (82 patients), Caucasian 2 deaths (6 patients), Hispanic 7 deaths (16 patients) Other 18 deaths (24 patients), BMI kg/m2 28 deaths (29.5 kg/m2), Bilateral infiltrates 74 deaths (121 patients), Respiratory 20 (41 patients), COPD 12 (22 patients), Asthma 2 (9 patients), Obstructive sleep apnea 8 (16 patients), Cardiovascular 70 (113 patients), Heart failure 19 (27 patients), Atrial fibrillation 10 (15 patients), Coronary Disease 19 (31 patients), Hypertension 69 (111 patients), Liver 4 (5 patients), Cirrhosis 4 (5 patients), Liver transplant 1 (2 patients), Renal 25 (35 patients), Chronic kidney disease 19 (26 patients), ESRD on dialysis 8 (11 patients), Kidney transplant 2 (2 patients), Other 46 (74 patients), Diabetes 45 (73 patients), HIV 2 (2 patients), Medications at admission: Antiplatelets 39 (60 patients), NSAIDS 5 (8 patients), ACEi/ARB 27 (49 patients).
Altschul et al. [19]	2,355	N/A	12.50%	46.68%	52.32%	Diabetes, Congestive heart failure, Chronic pulmonary disease, BMI (iqr), Myocardial infarction	Female - 294 patients age- years, mean SD 72.55, Black 252 patients, Asian -17 patients, Caicasian-81 patients, Other - Hispanic 211 patients, Diabetes 197 patients, Congestive Heart Failure 109 patients, Chronic Pulmonary Disease 61 patients, BMI (iqr) 28.2 kg/m2, Myocardial infarction 35 patients
Sharifpour et al. [20]	268	15.30%	9.70%	55.22%	44.40%	Diabetes, Hypertension	41 male and 26 female patients. 47 were Black and 20 belonged to other races. About 147 patients had comorbidities, Chronic Obstructive Pulmonary Disease (6 patients), Diabetes Mellitus (21 patients), Obesity (24 patients), Hypertension (55 patients), Stroke (11 patients), Coronary Artery Disease (14 patients), Smoking (24 patients), and Chronic Kidney Disease (12 patients). Mean age at death, days (SD) 71(13

Assessment of Methodological Quality and Data Extraction

Data from identified studies, selected through full-text screening, were retrieved to ascertain relevant patient demographics, including age, gender, race, duration of hospital stay, and comorbidities. Studies were re-evaluated for their quality based on their eligibility criteria. Any discrepancies encountered during the review process were addressed through consensus among the authors. The methodological quality of the chosen studies underwent independent assessment by the authors, utilizing operationalized quality criteria. The diagnostic criteria for identifying COVID-19 cases were grounded in clinical or diagnostic signs and symptoms. Comprehensive descriptions of the study samples, encompassing both clinical and demographic characteristics, were provided. All individuals diagnosed with COVID-19 were subsequently admitted for further observation and care. Prognostic factors were clearly defined, and outcomes were known for all or a significant majority of the patients. The assessment of outcomes was quantified through the CFR, and the reported results were adjusted for a minimum of two confounding variables/covariates to enhance the robustness of the findings. Each quality of the item was rated as 'yes' if the available data clearly and/or adequately described key characteristics of the item; otherwise, it was rated as 'no'. Items that did not provide any data were classified as 'not reported'. The response for each item was used to assess and report potential biases. Disagreements regarding specific study quality were resolved through consensus. Data analysis, including statistical methods, focused on four prognostic factors: the participants' gender, ethnicity, relevant comorbidities, and the viral load of COVID-19 within the individual (Table 3). This emphasis was based on previous studies showing an association between these factors and the outcome of case fatality in patients with COVID-19. Data were assessed for consistency, missing values, presence of outliers, and implausible values and documented during data extraction. Our analysis involved calculating case fatality rates based on the data reported by the original authors within the studies. We tracked two methods of adjusting for confounding variables, including stratification with a weighted measure average for point estimates. The second documented method for adjusting for confounding variables within studies was multivariate analyses. 

**Table 3:** Quality indicators of selected studies for prognostic factors of COVID-19 in hospitalized elderly patients.

Study	Setting	Sample described	Source of data	Diagnostic criteria	Prognostic factors defined	Outcome defined	Adjusted for confounders
Eboni et al. [12]	Single-center	Yes	Hospital records	Clinical	Yes	Yes	Yes
Bode et al. [13]	Single-center	Yes	Hospital records	Clinical	Yes	Yes	Yes
Garassino et al. [14]	Multi-center	Yes	Hospital records	Clinical	Yes	Yes	Yes
Wang et al. [15]	Multi-center	Yes	Hospital Records	Clinical	Yes	Yes	Yes
Choudhuri et al. [16]	Single center	Yes	Hospital records	Clinical	Yes	Yes	Yes
Berry et al. [17]	Multiple centers	Yes	Hospital records	Clinical	Yes	Yes	Yes
Chaudhary et al. [18]	Single Center	Yes	Hospital records	Clinical	Yes	Yes	Yes
Altschul et al. [19]	Single center	Yes	Hospital records	Clinical	Yes	Yes	Yes
Sharifpour et al. [20]	Multiple centers	Yes	Hospital records	Clinical	Yes	Yes	Yes

Results

**Figure 1:**
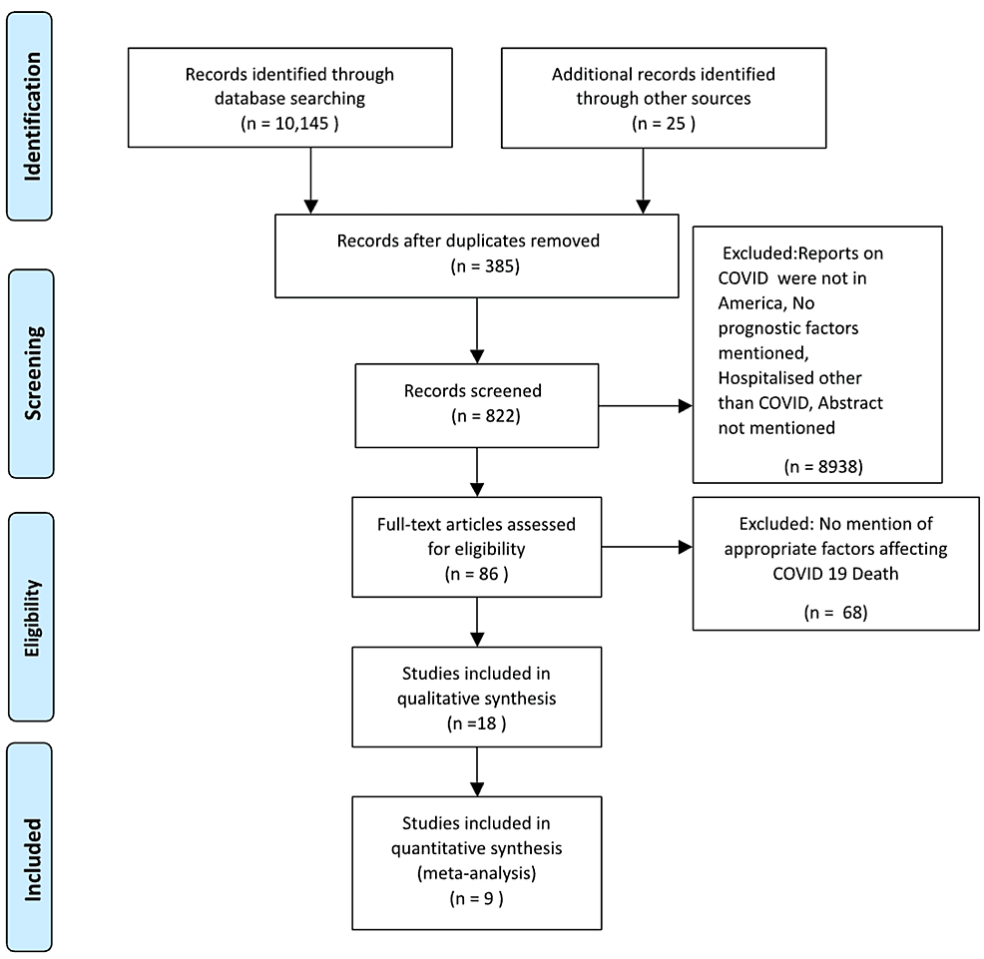
Prisma flow chart showing data extraction.

The literature search identified 10,170 articles related to the relevant exposure, outcomes, and study designs of interest. With the addition of PubMed filters (observational study, age: middle-aged + aged: 45+ years), 822 articles were retrieved for the initial screening of abstracts and titles. Out of these 822 citations, 86 papers remained for full-text review. Following the full-text review, 68 articles were excluded as they did not meet the inclusion criteria, leaving 18 articles for final inclusion. Additionally, nine more articles were excluded because the studies did not mention the prognostic factors affecting COVID-19 deaths in hospitalized patients. The nine studies that met our inclusion criteria comprised a total of 12,895 patients infected with COVID-19 (Figure 1). 

A variation in the quality of the study was found. Of the nine studies included, eight had a retrospective cohort design, and one had a longitudinal cohort design. Three out of nine studies were associated with race as their comorbidities, and the rest of the six studies included multiple underlying conditions like diabetes, body mass index (BMI), congestive heart failure, obesity, chronic obstructive pulmonary disease (COPD), etc., as their comorbidity factors. All nine studies have defined prognostic factors and outcomes. Overall, most studies found a significant association between COVID-19 and mortality as the prognostic factor. However, the direction of association was always the same; the more the severity of an underlying disease in COVID-19 patients, the greater the mortality rate (Table 4).

**Table 4:** Main findings from studies included in the prognostic factors for COVID-19 in hospitalized elderly patients. LOS: length of stay, ROC: receiver operating characteristic, AUC: area under the curve.

Study	Year	Design	Main Findings	Notes
Eboni et al. [12]	12	Retrospective	In a large cohort in Louisiana, 76.9% of the patients who were hospitalized with COVID-19 and 70.6% of those who died were black. Black race was not associated with higher in-hospital mortality than Caucasian race, after adjustment for differences in socio-demographic and clinical characteristics on admission.	Calculations for length of hospital stay were restricted to patients for whom the full length of stay could be determined. COVID–19–positive patients who identified themselves as Asian, American Indian or Alaska Native, Native Hawaiian or Pacific Islander, or Hispanic or who did not have a recorded race or ethnic group were excluded from the analysis.
Bode et al. [13]	13	Retrospective	COVID-19 patients with diabetes and/or uncontrolled hyperglycemia had a longer LOS and markedly higher mortality than patients without diabetes or uncontrolled hyperglycemia. Patients with uncontrolled hyperglycemia had a particularly high mortality rate.	Hyperglycemia is measured through blood glucose and HbA1C levels.
Garassino et al. [14]	14	Longitudinal	Univariable analyses revealed that being older than 65 years, being a current or former smoker, receiving treatment with chemotherapy alone, and the presence of any comorbidities were associated with increased risk of death. In multivariable analysis, only smoking history was associated with an increased risk of death.	Gender, age, smoking status, hypertension, and chronic obstructive pulmonary disease were included in the multivariable analysis.
Wang et al. [15]	15	Retrospective	While race was associated with a higher risk of infection, this paper did not find racial disparities in inpatient mortality, suggesting that outcomes in a single tertiary care health system are comparable across races. Identified key clinical features associated with reduced mortality and discharge. These findings could help to identify which COVID-19 patients are at the greatest risk of a severe infection response and predict survival.	
Choudhuri et al. [16]	16	Retrospective	SARS-CoV-2 Ct was found to be an independent predictor of patient mortality. After adjusting for age, gender, BMI, hypertension, and diabetes, an increased cycle threshold was associated with decreased odds of in-hospital mortality. Compared to the 4th Quartile, patients with Ct values in the 1st Quartile (Ct <22.9) and 2nd Quartile (Ct 23.0–27.3) had an adjusted odds ratio of in-hospital mortality of 3.8 and 2.6, respectively.	Prognostic was the Ct value, divided into quartiles for grouping samples and studying the significance of viral load as an indirect marker. Low Ct corresponded to higher viral load.
Berry et al. [17]	17	Retrospective	Advanced age was the strongest predictor of 40-day mortality. Relationship with advanced age was non-linear.	
Chaudhary et al. [18]	18	Retrospective	Blacks represented 64% of the population and had a likelihood of survival of 35% in comparison to 41% of the remaining population. Of the comorbidities, only respiratory, particularly asthma, were negatively associated with in-patient death. Respiratory comorbidities were present in 44% of subjects discharged alive versus 25% of in-patients who died. Asthma was present in 15% of subjects discharged alive versus 3% of in-patients who died.	Medication use did not differ by in-patient death.
Altschul et al. [19]	19	Retrospective	A ROC curve analysis was performed in the derivation cohort, and the novel COVID-19 severity score achieved an AUC of 0.824, indicating a good discrimination for patients with a higher risk of in-hospital mortality. The mortality rate for hospitalized patients was higher than the more commonly reported case-fatality rate that reflects the number of deaths per documented infection.	The mortality rates were slightly different between the training set (26.4%) and the testing set (22.4%). This is likely secondary to the temporal difference between the sets. The severity score remained valid in predicting in-hospital mortality.
Sharifpour et al. [20]	20	Retrospective	Results confirm that the median c-reactive protein value correlates with the severity of COVID-19 and is an independent predictor of mortality. Furthermore, results show that the rate at which c-reactive protein level rises during the first seven days of hospitalization could be used as a tool to predict disease progression and the need for early ICU transfer.	The maximum c-reactive protein value within the first seven days was less predictive of death.

Mortality

Gender: In nine studies, associated with mortality as the prognostic factor in COVID-19 patients, there were a total of 4,487 male cases and 4,533 female cases reported. Based on the data provided in (Table 5), there were 1,159 male deaths and 1,044 female deaths that were reported. We calculated the CFR individually of both males and females, which came out to be 25.8% and 23.0%, respectively.

**Table 5:** COVID-19 case fatality outcomes.

Patient Demographic / Variables	COVID 19 Deaths	COVID 19 Survivors	Total Cases	Case Fatality Rate %
Gender	2203	6817	9020	24.4
Male	1159	3328	4487	25.8
Female	1044	3489	4533	23.0
Black/African-American	582	1758	2340	24.9
Caucasian	806	1166	1975	40.8
Asian	84	371	455	18.5
Hispanic	395	1597	1992	24.7
Diabetes	700	1954	2654	26.4
Heart Conditions (Congestive Heart Failure, Hypertension, Coronary Artery Disease)	685	1616	2301	29.8
Chronic Obstructive Pulmonary Disease	79	142	221	35.7
Myocardial Infarction	35	102	137	25.5
Human Immunodeficiency Virus	40	113	153	26.1
Cancer (Thoracic Malignancies)	62	171	233	26.6
COVID Cycle Threshold (Viral Load)	Low CT = Higher Viral Load			
Q1 (<22.9)	109	154	263	41.4
Q2 (23.0 - 27.3)	84	179	263	31.9
Q3 (27.4 - 32.8)	43	217	260	16.5
Q4 (>32.9)	34	224	258	13.2

Race: In all nine studies, we came across different races and ethnicities and found out that there were four main races/ethnicities that were being talked about. These included Black/African-American, Caucasian, Asian, and Hispanic. The highest CFR obtained was in Caucasians which was 40.8%. However, no significant association between Caucasians and COVID-19 was mentioned. The total number of cases associated with COVID-19 in different ethnicities was 6,762 out of which, there were 1,867 deaths and 4,892 COVID-19 survivors, which is equivalent to the overall CFR of 27.6% (Table 5).

Comorbidities:Age >40 being the eligibility criteria for all our nine studies, makes COVID-19 patients very prone to underlying medical conditions like hypertension, diabetes, and heart conditions, among others. The highest CFR i.e., 35.7 % was associated with COPD as the comorbidity factor, as it is also associated with respiratory problems. The total number of cases reported with COVID-19 patients associated with different comorbidities was 5699 out of which, there were 1,601 deaths and 4,098 COVID-19 survivors, which is equivalent to the overall CFR of 28% (Table 5).

Discussion 

Summary of Main Findings

In this systematic review, we reported the prognostic factors for mortality in elderly patients hospitalized with COVID-19 infection. All participants in the review were COVID-19 inpatients diagnosed through reverse-transcriptase, polymerase chain reaction tests and above the age of 45 years in the North and South American continents. This study included 12, 895 patients who were hospitalized with the COVID-19 infection, and an overall 14.4% in-hospital case-fatality rate was demonstrated. The prognostic factor with the most significant association for increased risk of in-hospital mortality in the elderly was having a high viral load of the COVID-19 virus with a case-fatality rate of 41.4%. In addition, being of Caucasian ethnicity and having the comorbidity of chronic obstructive pulmonary disease (COPD) were also associated with higher rates of mortality within elderly hospitalized patients with case-fatality rates of 40.8% and 35.7%, respectively (Table 2).

Strengths and Limitations

The strength of this study is the addition of COVID-19 epidemiologic data based on a large and diverse cohort of patient population spanning through the entire geographical area of the Americas and the relatively large sample size of the studies included compared to the previous studies, with a lot of important demographic criteria of our patient population considered. In this study, case fatality was chosen as a measure of prognosis over the mortality rate, ensuring a better measure of disease severity putting into consideration that COVID-19 is an acute illness. It is also important to note that this is one of the first systematic reviews to focus on the prognostic factors associated with inpatient mortality in patients with COVID-19 in the Americas which has the highest mortality rate currently and the in-flow of clinical data is important to guide diagnostic, prognostic and treatment characteristics of the disease and is an essential part of understanding this illness. There were several limitations to this study. Firstly, there were only nine studies that were included in this research due to limitations in literature research involving newly evolved COVID-19. Secondly, not all studies reported the complete prognostic factors; Eboni et al., Bode et al., Garassino et al. [12-14] did not report on the percentage of male and female deaths due to COVID-19, also Altschul et al. and Chaudhary et al. [15-19] lacked reports on the percentage of male deaths. Thirdly, in the selective reporting of primary studies regarding the potential confounders, the possibility of non-survivors enrolled in studies dying of causes other than COVID-19 could not be excluded. Furthermore, there is a potential risk of bias related to the severity of the disease, including cases that did not have access to healthcare facilities. Finally, the studies included were based on structured data captured in the electronic medical record. Hence it was subject to the accuracy and completeness of electronic documentation by the care team.

Interpretation of Main Findings and Comparison with Other Studies

After assessing the results of all the nine studies (n= 12,895) that met our inclusion criteria, and the CFR associated with all the factors we took into consideration like gender, race, comorbidities, COVID-19 viral load, it was found that patients with chronic obstructive pulmonary disease (COPD) were discovered within this research study to possess the poorest prognosis in elderly hospitalized individuals with the COVID-19 infection. Research has shown that patients with underlying COPD possess a high risk for COVID-19 infection, due to their poor underlying lung reserve and increased expression of the angiotensin-converting enzyme 2 [15-21]. The upregulation of this enzyme predisposes patients to increased coronavirus infections, as the COVID-19 spike protein binds to this receptor to enter the cell. This relationship explains the increased risk and poor prognosis in active smokers and respiratory symptom exacerbations in patients with chronic obstructive pulmonary disease. These findings display the importance of smoking cessation and abstinence within elderly individuals who are prone to suffering from COPD. The elderly who are exposed to air pollutants also require caution and increased surveillance must also take precautions and be monitored to avoid adverse effects. Alqahtani et al. [4] conducted a systematic review of observational studies which demonstrated that COPD was associated with a 60% rate of mortality within hospitalized patients. This study does possess limitations in comparison to our study as their studies were pooled from China and the setting could affect outcomes due to differences in the healthcare systems in China and the United States of America. Rationalizing the increased rates of mortality in Caucasian patients compared to individuals of other ethnicities remains a difficult task as multiple contextual factors have to be taken into account. Whether this difference occurs due to the expression of the angiotensin-converting enzyme 2 (ACE2) receptor or other differences between the ethnicities at an organ level is a question that requires further research. This data is vital for determining health resource allocation as based on the information provided by the results of this study, Caucasian individuals should be viewed as high-risk groups for mortality when infected with the COVID-19 infection. The major limitation in determining whether these differences are legitimate is the lack of clinical studies to examine these effects, as our study is one of the first ones to examine this correlation with mortality (Table 4).

Implications for Clinicians

The studies included in this research not only reflect on outcomes of COVID-19-related hospital admission but also on various other determinants that critically reflect its implication on decision-making. Firstly, all studies included in this research predicted that there is a strong correlation between age in terms of mortality in COVID-19 hospitalized patients. This shows us that age plays a role in the severity of the disease. Older people should be treated with the first line of care and should be emphasized to be vaccinated earlier than other groups. In the study done by Berry et al. [15-17], they listed six features; age, tachypnea at presentation, hypoxia at presentation, history of hypertension, coronary artery disease, and renal insufficiency, that can be used in prognosticating an individual’s risk of dying within 40 days of COVID-19 related hospitalization. According to Choudhuri et al. [15-16], the use of a computed tomography (CT) scan can be an important indicator of measuring the viral load and mortality within hospitalized individuals. The studies also focused on race and its relation to mortality in COVID-19 hospitalized patients. Berry et al. [15-17] highlighted that age was significantly related to death only among Black subjects, who were marginally younger, compared with subjects in the Caucasian/Hispanic/other race/ethnic group. Similar findings were noted in the study by Eboni et al. [12]. They reported that the racial difference in the clinical presentation can be due to a long wait to access care among black patients resulting in more severe illness on presentation to health care facilities. However, these findings were in contrast with the study by Wang et al. [15], where they found no significant association between race and clinical outcome after adjusting for underlying covariates. Hence the evidence within this area needs to be strengthened in future research studies. The studies also raised a few important questions that need to be addressed in future research implications. Bode et al. [13] pointed out the need for a correlation between metabolic disturbances e.g., diabetes, in terms of mortality from COVID-19 deaths, as it was one of the prognostic factors in their study. Garassino et al. directed the need for strengthening of evidence in terms of intubation in hospitalized patients and more aggressive care in patients with cancer and COVID-19 survival [20].

Unanswered Questions and Future Research

Due to the heterogeneous nature of results, thresholds during hospitalization that may facilitate risk stratification and prognostication are still poorly understood on which patients are at the greatest risk. Ongoing clinical features tracked with disease progression were found to be an independent predictor of in-patient mortality. However, further study is needed on how to best clinically utilize such information given the result variation due to specimen quality, phase of the disease, and the limited discriminative ability.

## Conclusions

In the current study, prognostic factors for COVID-19 mortality among a large sample of hospitalized elderly patients in North and South American continents were evaluated. Having a high viral load of the infection, being of Caucasian ethnicity and underlying pulmonary comorbidities such as COPD were independent prognostic factors for mortality among these patients. Extra attention should be paid to these factors, and further research should be undertaken to determine the underlying mechanisms behind these effects.
